# X-Linked Signature of Reproductive Isolation in Humans is Mirrored in a Howler Monkey Hybrid Zone

**DOI:** 10.1093/jhered/esaa021

**Published:** 2020-07-29

**Authors:** Marcella D Baiz, Priscilla K Tucker, Jacob L Mueller, Liliana Cortés-Ortiz

**Affiliations:** 1 Department of Ecology & Evolutionary Biology, University of Michigan, Ann Arbor, MI; 2 Department of Human Genetics, University of Michigan, Ann Arbor, MI; 3 Department of Biology, Pennsylvania State University, University Park, PA

**Keywords:** speciation, genomic clines, sex chromosomes, gene flow, primates

## Abstract

Reproductive isolation is a fundamental step in speciation. While sex chromosomes have been linked to reproductive isolation in many model systems, including hominids, genetic studies of the contribution of sex chromosome loci to speciation for natural populations are relatively sparse. Natural hybrid zones can help identify genomic regions contributing to reproductive isolation, like hybrid incompatibility loci, since these regions exhibit reduced introgression between parental species. Here, we use a primate hybrid zone (*Alouatta palliata* × *Alouatta pigra*) to test for reduced introgression of X-linked SNPs compared to autosomal SNPs. To identify X-linked sequence in *A. palliata*, we used a sex-biased mapping approach with whole-genome re-sequencing data. We then used genomic cline analysis with reduced-representation sequence data for parental *A. palliata* and *A. pigra* individuals and hybrids (*n* = 88) to identify regions with non-neutral introgression. We identified ~26 Mb of non-repetitive, putatively X-linked genomic sequence in *A. palliata*, most of which mapped collinearly to the marmoset and human X chromosomes. We found that X-linked SNPs had reduced introgression and an excess of ancestry from *A. palliata* as compared to autosomal SNPs. One outlier region with reduced introgression overlaps a previously described “desert” of archaic hominin ancestry on the human X chromosome. These results are consistent with a large role for the X chromosome in speciation across animal taxa and further, suggest shared features in the genomic basis of the evolution of reproductive isolation in primates.

Recent advances in DNA sequencing have opened the opportunity to explore the genetics of speciation in non-model organisms, allowing for genome-wide identification of loci associated with reproductive isolation and their comparison among divergent lineages. Results from this research corroborate early observations (e.g., Coyne and Orr 1989) that sex chromosomes seem to be important drivers of reproductive isolation in many animal taxa (e.g., birds: Sætre et al. 2003; Carling and Brumfield 2008; Irwin 2018; *Drosophila*: Presgraves 2008; *Mus*: Good et al. 2008a, 2008b, 2010; Janoušek et al. 2012; fish: Kitano and Peichel 2012), including humans (Sankararaman et al. 2014, 2016). Some contemporary humans with ancestors from outside of Africa carry as much as ~5% archaic DNA as a result of ancient admixture with Neanderthals and Denisovans (Sankararaman et al. 2014, 2016). However, there are many regions of reduced ancestry from archaic hominins in modern human genomes (i.e., “deserts” of archaic ancestry), which are highly enriched on the X chromosome (Sankararaman et al. 2014, 2016). Further, Neanderthal Y chromosome sequence has not been discovered in contemporary human genomes (Mendez et al. 2016). These findings imply that X and Y chromosomal regions may have been involved in reproductive isolation during the time of hybridization, and thus, archaic ancestry was rapidly purged by selection acting on unfit hybrids, limiting introgression on sex chromosomes. In hybrid flies and mice, loci underlying hybrid male sterility disproportionately map to the X chromosome, consistent with a large role of the X chromosome in speciation (e.g., Presgraves 2008; Turner and Harr 2014).

Because the genomic signature of ancient genetic exchanges is all that is left of our archaic cousins, it is not possible to directly investigate the mechanisms that may have lead to restricted gene flow in certain genomic regions while allowing exchange in others. We address this here by identifying genomic regions that are consistent with the signature of reproductive isolation (i.e., “barrier loci”) between 2 contemporary primate species that form a natural hybrid zone and ask whether deserts of archaic ancestry on the human X chromosome are unique to hominids or shared with other primates. Natural hybrid zones offer unique opportunities to identify barrier loci because reproductive isolation between the parental taxa is still incomplete. In hybrid zones, novel combinations of alleles in hybrids are tested by selection (Dobzhanksy 1936; Muller 1942). Recombination shuffles composite genomes with many generations of backcrossing, and loci that underlie reproductive isolation are expected to have restricted introgression as a result of selection against unfit hybrids carrying incompatible alleles, while neutral and adaptive alleles are free to introgress between species (Barton and Hewitt 1985; Gompert and Buerkle 2011).

The howler monkey hybrid zone (*Alouatta palliata* × *Alouatta pigra*) in Mexico (Cortés-Ortiz et al. 2007) has recently developed into a model for comparison of natural populations to laboratory-derived and other traditional model systems (e.g., *Mus*, *Homo*) that have been the subject of intensive speciation genomics research. A genome assembly was recently developed for *A. palliata*, opening the possibility for detailed investigation of fine-scale variation contributing to reproductive isolation and other evolutionary processes. Further, the *Alouatta* system is the first contemporary primate system in which the genomic architecture of reproductive isolation has been investigated (Baiz et al. 2019; Cortés-Ortiz et al. 2019), thus due to its relatively close phylogenetic proximity, it serves as a model that may yield insight to processes responsible for generating similar patterns observed in human genomes.

*Alouatta palliata* and *A. pigra* diverged ~3 MA (Cortés-Ortiz et al. 2003), and the contact zone is likely the result of secondary contact after periods of isolation and expansion (Cortés-Ortiz et al. 2003; Ellsworth and Hoelzer 2006; Ford 2006). We previously analyzed introgression in this system with a limited number of loci and found differential introgression for autosomal markers (i.e., some had reduced introgression, others had neutral, or directional introgression), but markers on the X and Y chromosomes had restricted introgression (Cortés-Ortiz et al. 2019). This observation is consistent with a role for the sex chromosomes in reproductive isolation in this system.

Due to the limited number of markers analyzed in the previous study, we were unable to determine the extent of reduced introgression across the X chromosome. Here, we used comparative whole-genome re-sequencing to identify X chromosome sequence in the *A. palliata* genome assembly. We then used genomic cline analysis with population-level reduced-representation genome sequence data to explicitly test for reduced introgression of X-linked compared to autosomal SNPs and to quantify the pattern of introgression along the X chromosome. We compare our results to signatures of archaic introgression in the human genome to ask whether there is a shared component of the genomic architecture of speciation in primates.

## Materials and Methods

Since the *A. palliata* genome assembly is a draft de novo assembly for which contigs have yet to be assigned to chromosomes, we first performed a mapping experiment using whole-genome re-sequencing data from 2 males and 2 females to identify X-linked contigs. To do this, we analyzed differences in contig-specific sequencing coverage between female and male *A. palliata* individuals with the assumption that X-linked contigs will show significantly greater coverage in females than males because, in XY species, females have 2 copies of the X chromosome and males have 1 copy (we refer to these as “female-biased” contigs). In comparison, autosomal contigs should have equal coverage in males and females (“unbiased” contigs). This method has been used in several study systems to confidently identify sex chromosome sequences (e.g., Chen et al. 2012; Carvalho and Clark 2013; Gamble et al. 2015; Vicoso and Bachtrog 2015; Bracewell et al. 2017; Mongue et al. 2017). Supplementary Figure S1 illustrates an overview of our strategy.

### Whole-Genome Re-Sequencing

Between 2001 and 2012, we obtained blood samples from 4 wild *A. palliata* individuals (2 males and 2 females) sampled in Veracruz, Mexico (Supplementary Table S1) and stored them in lysis buffer at −80 °C. We extracted genomic DNA using the QIAGEN DNeasy tissue kit (Qiagen Inc., Valencia, CA) as described in Baiz et al. (2019). Sex was determined in the field by visual assessment and verified using genetic data by amplifying known genes on the sex chromosomes (following Cortés-Ortiz et al. 2019). Specifically, we amplified the Y-linked *SRY* locus to verify the presence of a PCR product for males and absence of a PCR product for females and genotyped an X-linked microsatellite locus to verify the hemizygous genotype for males (Supplementary Table S1).

Our libraries for whole-genome sequencing were generated and sequenced by the Advanced Genomics Core at the University of Michigan. For each sample, libraries were constructed using the Swift Biosciences PCR-Free DNA Library Kit with a target insert size of 350bp following the manufacturer’s protocol. Libraries were multiplexed and sequenced in a single lane using Illumina HiSeq 4000 to obtain 150bp paired-end reads.

We obtained between ~54 M–123 M reads per individual from the sequencer (Supplementary Table S1). We used *Trim Galore!* v0.4.2 (http://www.bioinformatics.babraham.ac.uk/projects/trim_galore/) to trim adapters and low-quality bases (Q < 20) from raw reads and retained only read-pairs where each read was ≥100bp in length after trimming.

### Detection of X-Linked Contigs

To avoid false identification of X-linked contigs due to differences in sequencing coverage among males and females, we subsampled our post-trimmed fastq files to standardize the number of reads across individuals using *seqtk* v1.2 (https://github.com/lh3/seqtk) before mapping by randomly sampling 50 million read pairs per individual. We used these subsampled files as input for sequence alignment.

Because short read data can potentially map to multiple genomic locations due to the expansion of repetitive sequence, we generated a repeat-masked version of the reference *A. palliata* genome assembly (PVKV00000000) in order to map reads to unique sequences. To do this, we used RepeatMasker v4.0.6 (Smit et al. 2013) on *A. palliata* contigs 1 kb and larger (*N* = 96 654, 87% of the assembly sequence) using the “primates” RepeatMasker repeat library (i.e., a library of known primate repeats) to perform a low-sensitivity search (option –qq).

We then used bwa-MEM (Li 2013, https://arxiv.org/abs/1303.3997, last accessed 21 May 2019) to align the subsampled reads of our 4 *A. palliata* individuals to the repeat-masked *A. palliata* genome assembly. For each individual, we used SAMtools idxsats (Li et al. 2009) to count the number of reads mapped to each masked contig. We then used exact tests in edgeR (Robinson et al. 2010) to detect contigs for which there was a significant difference in read counts between the sexes, where X-linked contigs are expected to have an average male-to-female log_2_ fold-change (log_2_FC) of −1 (due to the presence of 2 X chromosomes in females and only 1 in males) and autosomal contigs are expected to have a log_2_FC of 0. For this analysis, we excluded contigs with low counts across samples as they provide limited power to detect significant differences between groups. Thus, we only retained contigs where 2 or more individuals had a count per million (CPM) >0.2, corresponding to ~15 reads (*N* = 78,493 contigs).

### Validation of X-Linkage

We aligned *A. palliata* repeat-masked contigs for which we detected sex-differences in read count to the marmoset (CalJac3) and human (hg38) genome assemblies to identify orthologous regions. We used these results as a second line of evidence for X-linkage of female-biased contigs since X chromosome sequence is highly conserved across mammals. To do this, we downloaded the masked version of each assembly from the University of California Santa Cruz Genome Browser and used a custom script (available at https://github.com/baizm/Xchr_introgression) to remove scaffolds that have not yet been assigned to any chromosome (i.e., sequences with a header containing “chrUn”). We then used *lastz* v.1.04.00 (a program designed for efficient alignment of long genomic sequences, Harris 2007) to align the repeat-masked *A. palliata* contigs to each repeat-masked assembly, requiring at least 50% of the query to be included in the alignment block (--coverage = 50) and using a distance of 20bp between potential seeds (--step = 20). To assess the ability of our method to detect X-linked sequence, we also aligned a set of 2,288 unbiased, likely autosomal contigs for comparison (i.e., the same as the number of female-biased contigs), randomly chosen from the list of contigs that did not have a significant difference in read counts between the sexes.

For female-, male-, and unbiased *A. palliata* contigs, a larger proportion aligned to the marmoset genome (>55% for each contig type) than to the human genome (Supplementary Figure S2). For male-biased and unbiased *A. palliata* contigs, the proportion that aligned to autosomes (~94–99%) or sex chromosomes (~1–5%) was similar for the marmoset and human genome (Supplementary Figure S2). Further, the majority (74%) of the female-biased contigs that aligned to the human X chromosome also aligned to the marmoset X chromosome. This pattern is not surprising, given that the divergence time between *Alouatta* and *Homo* is greater than between *Alouatta* and *Callithrix* (Perelman et al. 2011). Thus, we considered female-biased contigs that aligned to the marmoset X chromosome to be X-linked for *Alouatta* in this study.

We used qPCR to test for the expected 2-fold higher amplification of putative X-linked regions in female individuals compared to male individuals. We randomly selected 5 putative X-linked contigs and 1 putative autosomal contig to serve as a normalizing sequence assuming 1:1 amplification between the sexes. The 5 putative X-linked contigs were selected from *A. palliata* female-biased contigs that mapped to the marmoset X chromosome. The putative autosomal contig was randomly selected from *A. palliata* unbiased contigs that mapped to a marmoset autosome. The putative X-linked contigs mapped to positions that spanned the length of the marmoset X chromosome (between X:6.6 and X:104 Mb) and the putative autosomal contig mapped to marmoset chromosome 1 (Supplementary Table S2). Details of our qPCR strategy are outlined in Supplementary Methods.

### ddRADseq and SNP Calling

**Figure 1.**
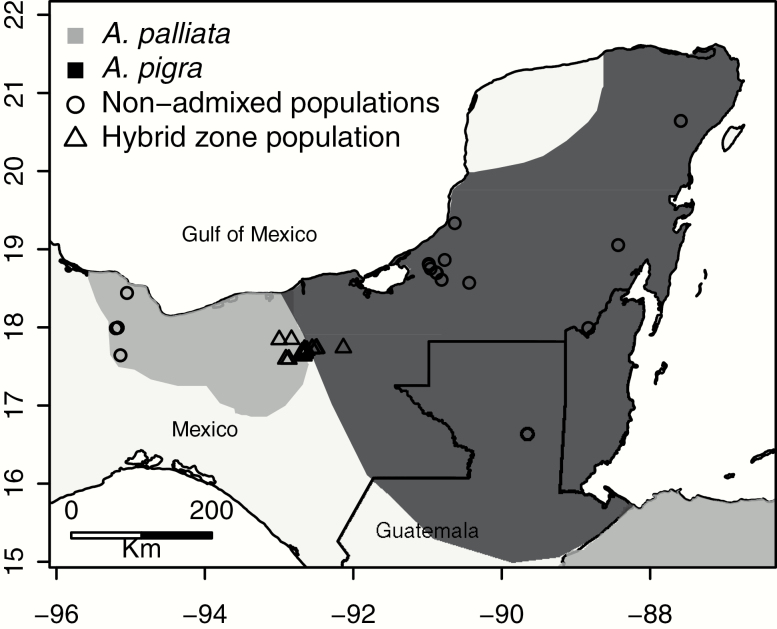
Map of sampling sites used in this study. The distribution ranges of *Alouatta palliata* and *Alouatta pigra* are in light gray and dark gray, respectively (downloaded and modified from IUCN, http://www.iucnredlist.org). Circles represent sampled localities where only one species occurs (i.e., non-admixed populations) and triangles represent sampled localities from the hybrid zone, where admixed individuals occur.

We performed reduced-representation sequencing on a geographically broad sample of wild individuals from the allopatric ranges of *A. palliata* and *A. pigra* and from the hybrid zone (see Baiz et al. 2019 for details) (Figure 1) and mapped sequence reads to the non-masked *A. palliata* assembly to generate genotype data for genomic cline analysis to test for reduced introgression of the X chromosome. Because the X chromosome is hemizygous in males and X-linked SNPs will appear to be homozygous, biasing genomic cline estimates, we only included sequence data from female individuals in genomic cline analyses to avoid bias in our X chromosome-autosome comparison of differential introgression. This included 88 female individuals, 48 of which were sampled from the hybrid zone in Tabasco, Mexico, 17 from the allopatric range of *A. palliata* and 23 from the allopatric range of *A. pigra* (Figure 1), which we included in our genomic cline analysis. All allopatric individuals included here have been previously shown to be non-admixed (Baiz et al. 2019). We used double digest restriction site-associated DNA sequencing (ddRADseq, Peterson et al. 2012) to generate reduced-representation genome sequence data for these individuals, as described in Baiz et al. (2019).

We retained only biallelic SNPs with a minor allele frequency of at least 0.05, a minimum mean read depth of 12 across all individuals, and sites present in 14 or more individuals in either parental population. To reduce the effects of linkage among markers, and because previous analyses indicated that SNPs on the same *Alouatta* contig generally show consistent patterns of introgression (Baiz et al. 2019), we retained only 1 SNP at random per contig. This resulted in 10 353 SNPs used in the genomic cline analysis. The combined length of the *Alouatta* contigs containing SNPs used in our analysis represents ~39% of the *A. palliata* genome assembly. We considered X-linked SNPs to be those on female-biased contigs that mapped to the marmoset X chromosome (*N*_SNPs_ = 97) and autosomal SNPs to be those on contigs that had no significant difference in read counts between the sexes (*N*_SNPs_ = 10 256). The set of X-linked and autosomal SNPs represent approximately equal genotyping densities on female-biased (1.9 × 10^–5^ SNPs/Mb) and unbiased contigs (8.4 × 10^–6^ SNPs/Mb). All filtering steps were carried out using *bcftools* v.1.3.1, *vcftools* 0.1.14 (Danecek et al. 2011), and custom scripts (available at https://github.com/baizm/Xchr_introgression).

### Genomic Cline Analysis

To analyze the pattern of introgression for X-linked and autosomal SNPs, we calculated genomic clines for each locus using *bgc* (Gompert and Buerkle 2011; Gompert and Buerkle 2012), as described in Baiz et al. (2019). This analysis uses Markov chain Monte Carlo to estimate cline parameters in a Bayesian genomic cline model and identify outlier loci that are more or less likely than the genome-wide average (assumed to be neutral) to introgress between parental populations. Two cline parameters are used to summarize the amount (β) and direction (α) of introgression. Loci associated with reproductive isolation are expected to have reduced introgression (β > 0), while loci with increased introgression (β < 0) may be candidates for adaptive introgression. Loci with a shift in cline center reflect directional movement of alleles into *A. palliata* (excess *A. pigra* ancestry, α > 0) or movement into *A. pigra* (excess *A. palliata* ancestry, α < 0).

We ran *bgc* analyses using the genotype uncertainty model and ran 5 independent chains, each with a burn-in of 30,000 for 50,000 steps, and thinned samples by 20. We then merged outputs and identified outlier SNPs for both β and α from MCMC output as SNPs with a 95% credible interval that does not overlap zero.

### Comparing Introgression of the X Chromosome Versus Autosomes

To test if X-linked SNPs have a distinct pattern of introgression, we tested for significant differences in cline parameters between X-linked and autosomal SNPs using permutation tests in R. We constructed 10 000 permuted datasets from the autosomal data by sampling without replacement from the distribution of cline parameter point estimates for both α and β. For each permuted dataset, we sampled 97 of the 10 256 autosomal SNPs without replacement, so comparisons were made using a sample size equal to the set of X-linked SNPs (*N* = 97). We compared the mean of the observed cline parameter for X-linked SNPs to the mean cline parameter of each permuted autosomal dataset and considered the pattern of introgression for X-linked SNPs to be distinct if the observed mean exceeded the mean in >95% of the permuted datasets.

### Genomic Basis of Non-neutral Introgression of the X Chromosome

To identify genes on contigs containing SNPs with non-neutral introgression, we queried the marmoset X chromosome for genes in homologous regions using *biomaRt* v2.36.1 (Durnick et al. 2005, 2009). To do this, we input the marmoset alignment block coordinates for alignments of each X-linked *bgc* outlier contig expanded by 500kb on both ends to obtain marmoset genes within each region. We also report human gene homologs within each region, as the gene annotation of the human genome assembly is more complete than for the marmoset assembly.

To determine if the previously observed “deserts” of archaic hominin ancestry in the human genome are homologous to the regions of reduced introgression we observed here for *Alouatta*, we plotted cline parameter estimates along the human X chromosome for X-linked contigs in our *bgc* dataset that mapped to the human X chromosome using our alignment criteria (*N* = 52) with *karyoPloteR* v1.6.2 (Gel and Serra 2017). All R-based analyses were conducted using R v3.5.1.

## Results

### X-Linked Contigs in the *A. palliata* Genome

Upon mapping our re-sequencing data to the masked version of the *A. palliata* assembly we generated, we found that read counts for most contigs (97%) were not significantly different between males and females (i.e., “unbiased” contigs), suggesting they are autosomal (Table 1). Thus, we used this set of contigs to call autosomal SNPs for our genomic cline analyses. We detected 2,288 contigs with read counts significantly greater in females than in males (i.e., “female-biased” contigs). Read counts for these contigs were, on average, 2-fold greater in females than in males (mean log_2_FC of ~1), as expected for X-linked sequence. We also detected 390 contigs with higher read counts in males than in females (i.e., “male-biased” contigs). Log_2_FC was much more variable for these male-biased contigs (Table 1, Supplementary Figure S3). Because the reference genome was generated by sequencing a female individual (Jeremy Johnson, personal communication), it should not contain sequence unique to the Y-chromosome, and since we were only interested in discerning putatively X-linked from putatively autosomal sequences, we dropped these ambiguous male-biased contigs from further analyses.

**Table 1.** Summary of mapping experiments to identify X-linked contigs in the *Alouatta palliata* assembly

Read count bias	N contigs (%)	Mean log_2_FC (M:F) ± SD	N contigs mapped to marmoset (%)
Unbiased	75 815 (97%)	−0.003 ± 0.30	1392^a^ (60.1%)
Female	2288 (2.9%)	−0.992 ± 0.38	1077 (47.1%)
Male	390 (0.5%)	2.129 ± 1.53	179 (45.9%)

N contigs = number of contigs detected to be biased or unbiased in edgeR, Mean log_2_FC = mean log_2_-fold-change of read counts for male data relative to female data.

^a^The number of contigs mapped to marmoset for unbiased contigs was 2288, randomly chosen to match the sample size of female-biased contigs.

To corroborate X-linkage of *A. palliata* assembly contigs, we took advantage of the high degree of conservation of the mammalian X chromosome (Ohno 1967; Quilter et al. 2002; Raudsepp et al. 2004; Murphy et al. 2007; Rodríguez Delgado et al. 2009) and used sequence homology of our putative X-linked regions to the marmoset X chromosome. We also used quantitative PCR (qPCR), to validate X-linkage of *A. palliata* sequences. Of the set of 2,288 *A. palliata* female-biased contigs, 1,077 could be mapped to the marmoset genome using our mapping criteria (Table 1). Of these contigs, 811 (75.3%) mapped to the marmoset X chromosome, 277 (25.7%) mapped to marmoset autosomes, and 1 mapped to the marmoset Y chromosome. This enrichment of hits to the marmoset X chromosome is consistent with the results of our comparative read count analysis (see above), indicating that these regions are likely to be X-linked in *A. palliata*. Comparatively, a much smaller proportion of the unbiased contigs (3.4%) and male-biased contigs (1.1%) mapped to the marmoset X chromosome while the majority mapped to autosomes (Supplementary Figure S2). Further, log_2_FC for the subset of the 811 female-biased contigs that mapped to the marmoset X chromosome was less variable and closer to the expected −1 for X-linked sequences compared to the larger pool of 2288 female-biased contigs (Supplementary Figure S4). Thus, this set of 811 female-biased contigs that mapped to the marmoset X chromosome constitutes our set of validated X-linked regions used in further analyses.

As proof of concept, we randomly chose 5 of the 811 female-biased contigs that mapped to the marmoset X chromosome to confirm 2-fold amplification in females relative to males (as expected for X chromosome sequence) using qPCR. Consistent with this, mean fold-change was 2.19 ± 0.49 (Table 2). These observations are consistent with high conservation of the X chromosome in mammals (Ohno 1967; Quilter et al. 2002; Raudsepp et al. 2004; Murphy et al. 2007; Rodríguez Delgado et al. 2009) and further corroborate our method of identifying X-linked versus autosomal sequence.

**Table 2.** qPCR validation of 5 *Alouatta palliata* X-linked contigs

Contig	∆∆Ct	Fold-change
26402	0.92	1.89
35197	1.55	2.94
92787	1.14	2.20
118733	1.17	2.25
60023	0.73	1.65

∆∆Ct is relative quantification of template DNA for each female-biased contig (i.e., “gene-of-interest”) compared to an unbiased (i.e., autosomal) marker (“normalizing gene”). The autosomal marker used is on *A. palliata* contig 84001.

Because a portion of our female-biased contigs mapped to autosomes, it is possible that we detected X chromosome sequence in *A. palliata* that is not shared with other primates (i.e., lineage-specific translocations to the X chromosome). To explore this, we looked at the mapping positions of the 277 female-biased contigs that mapped to marmoset autosomes and compared them to the mapping positions of male-biased and unbiased contigs for the autosomes with the most hits. After the X chromosome, chromosome 7 had the highest number of hits for female-biased contigs (Supplementary Figure S5A). However, chromosome 7 also had the highest number of hits for both male-biased and unbiased contigs (Supplementary Figure S5A), and for all contig types, the hits were clustered around positions 29.6 MB and 74.3 MB (Supplementary Figure S5B). Similarly, chromosome 21 had the third highest number of hits for female-biased contigs, high mapping numbers for male-biased and unbiased contigs, and a clustering of mapping positions around 17.7 MB (Supplementary Figure S5C). These mapping positions are not unique to sex-biased contigs, which may be due to multiple factors, including misassembly. This may also indicate that these sequences are not unique to the sex chromosomes. Thus, we dropped female-biased contigs that map to marmoset autosomes and male-biased contigs from further analyses to account for this uncertainty.

### Distinct Introgression of X-Linked SNPs

Genomic cline parameters for most SNPs were consistent with neutral introgression, but we detected 211 outliers with a non-neutral amount of introgression (β) and 746 outliers with a non-neutral direction (α) of introgression (Table 3, Supplementary Figure S6). Among outlier autosomal SNPs, the majority had increased introgression (β < 0, *N* = 194) and excess ancestry from *A. pigra* (α > 0, *N* = 470). On the other hand, outlier X-linked SNPs had reduced introgression (β > 0, *N* = 2) and excess ancestry from *A. palliata* (α < 0, *N* = 3).

**Table 3.** Number of X-linked (type = X) and autosomal (type = A) SNPs with neutral (zero) and extreme introgression (outliers)

Cline parameter	Type	Negative outlier	Zero (neutral)	Positive outlier
β	X	0	95	2
	A	194	10 047	15
	Total	194	10 142	17
α	X	3	94	0
	A	273	9513	470
	Total	276	9607	470

The cline parameter β is a measure of the amount of introgression, where negative outliers have increased introgression (β < 0) and positive outliers have reduced introgression (β > 0). The cline parameter α measures the direction of introgression where negative outliers (α < 0) have excess *Alouatta palliata* ancestry and positive outliers (α > 0) have excess *Alouatta pigra* ancestry.

**Figure 2.**
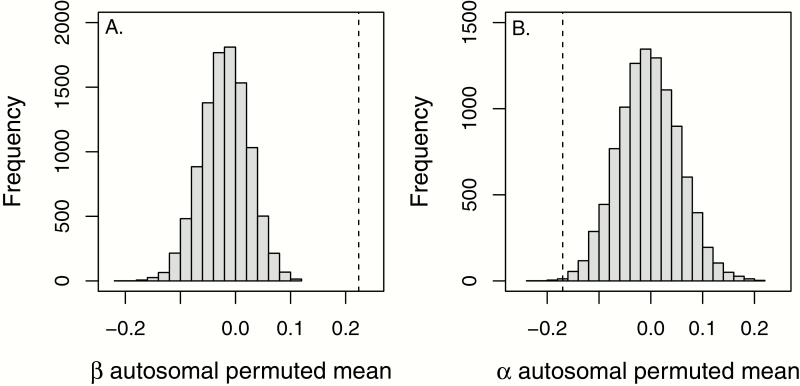
Histogram of means of 10 000 permuted autosomal SNP datasets (gray bars) for (**A**) the amount of introgression (β) and (**B**) the direction of introgression (α). In each case, the vertical dashed line is the observed mean for all X-linked SNPs, which is more extreme than the mean of the permuted data set in >95% samples indicating X-linked SNPs have a distinct pattern of introgression with respect to both cline parameters. Reduced introgression is indicated by β > 0 and increased introgression by β < 0. Excess *Alouatta pigra* ancestry is indicated by α>0 and excess *Alouatta palliata* ancestry by α<0.

Our permutation tests using all SNPs indicate that cline parameters are more extreme for X-linked than for autosomal SNPs (Figure 2), suggesting a distinct pattern of introgression for the X chromosome. For X-linked SNPs, the amount of introgression was significantly reduced compared to autosomal SNPs (mean β _X_ = 0.22, mean β _A_ = −0.02, *P* < 0.001) and the direction of introgression was more negative (mean α _X_ = −0.17, mean α _A_ = −0.003, *P* < 0.001), indicating excess ancestry from *A. palliata*, consistent with the signal for outlier loci.

### Shared Genomic Signature of Reduced X Chromosome Introgression

To identify regions of the *Alouatta* X chromosome associated with SNPs exhibiting non-neutral introgression, we used homology of *Alouatta* contigs to the marmoset and human X chromosome. After adding 500kb to each end of the alignment block within the marmoset X chromosome for alignments of *Alouatta* contigs containing outlier SNPs, 2 regions with excess *A. palliata* ancestry overlapped in the marmoset assembly (X: 46475367: 47494965, X:47487523:48488951). Thus, we report this as a single region (region 1), which had the greatest gene content in comparison to the other 3 X-chromosomal regions containing outlier loci (Table 4). The other outlier contig with excess *A. palliata* ancestry mapped to the long arm of the marmoset X chromosome (region 2). The 2 contigs containing SNPs with reduced introgression mapped more distally (regions 3 and 4) (Table 4).

**Table 4.** Alignment positions to the marmoset genome and gene content of X-linked *Alouatta palliata* contigs containing SNPs with non-neutral introgression

Contig	Length (kb)	CalJac3	Region	N genes	Cline parameter estimate
49400	23.4	X:46475367:47494965	1	50	α = −0.94
151667	1.4	X:47487523:48488951			α = −0.69
30014	54.1	X:67467391:68496613	2	4	α = −0.97
32694	48.1	X:113940392:114968526	3	3	β = 0.84
54333	18.8	X:135586677:136602592	4	3	β = 0.88

CalJac3 = the coordinates of the biomaRt query which includes an extension of 500kb on each end of the alignment block, Region = orthologous region as referred to in the text (e.g., “region 1”), N genes = the number of genes within each region, and Cline parameter estimate = *bgc* cline parameter estimate, where α is direction and β is the amount of introgression.

**Figure 3.**
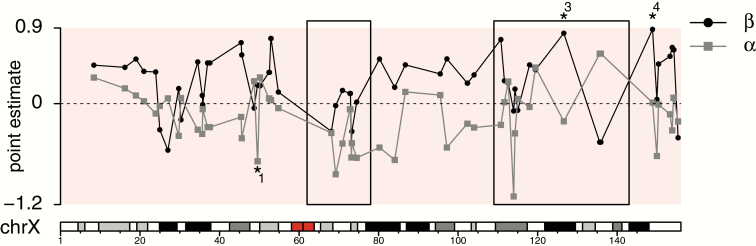
Cline parameter estimates for SNPs within *Alouatta* contigs that mapped to the human X chromosome (“chrX,” bottom). Asterisks denote outlier SNPs with non-neutral introgression and numbers correspond to the regions in Table 4. Note that region 2 did not map to the human X chromosome. The direction of introgression is measured by α (gray) and the amount of introgression is measured by β (black). The 2 previously described “deserts” of archaic ancestry (Sankararaman et al. 2016) are enclosed in boxes. Shaded regions along the human X chromosome are cytobands and the centromere is represented in red.

Of the 97 X-linked contigs represented in our SNP dataset, 52 mapped to the human X chromosome. The 45 remaining contigs did not map to the human genome using our mapping criteria. Of the 2 X-linked contigs containing SNPs with reduced introgression, 1 (region 3) mapped to a position within one of the previously described human “deserts” for ancestry from both Neanderthals and Denisovans (Sankararaman et al. 2016) (Figure 3). The other contig with reduced introgression (region 4) mapped just distally, but outside of the same desert. Finally, a region 1 contig with excess *A. palliata* ancestry mapped to the proximal end of the short arm of the human X chromosome. Only one of these regions (region 2) did not map to the human X chromosome. These results are consistent with our mapping analysis using the marmoset X chromosome (Table 4), and the expected high degree of conservation in X chromosome sequence among primates.

## Discussion

Here, we identified extensive X-chromosomal sequence in the *A. palliata* genome assembly and used it to test for differential introgression of the X chromosome in the *A. palliata* × *A. pigra* hybrid zone. Loci with reduced introgression were disproportionately represented on the X chromosome compared to autosomes, and one outlier locus overlaps a previously identified desert of archaic ancestry on the human X chromosome, suggesting a shared genomic architecture of reproductive isolation in primates. We also detected a conflicting signal of directional introgression, where autosomal loci had excess ancestry from *A. pigra* whereas X-linked loci had excess ancestry from *A. palliata*.

### Discovery of X Chromosome Sequence in *A. palliata* and Inherent Limitations

This is the first study to identify extensive, contiguous sex chromosome sequence in *A. palliata*. Using whole-genome re-sequencing data, we identified 811 contigs in the *A. palliata* genome assembly to be X-linked based on greater mapped read counts in females compared to males and alignment to the marmoset X chromosome. Thus, it is very likely these sequences are indeed on the *Alouatta* X chromosome. This approach allowed us to test for differential introgression of X-linked versus autosomal SNPs using our reduced-representation dataset.

We were very conservative in our mapping approach to ensure a low likelihood of identifying false positive X-chromosomal regions by taking several precautions. First, because we re-sequenced only 2 male and 2 female individuals, we standardized the number of raw reads to ensure our results were not influenced by spurious differences in sequencing coverage among individuals. We also limited our X chromosome mapping analyses to non-repetitive sequence by repeat masking the genome. This step ensured that read count differences were likely caused by chromosomal differences between males and females rather than copy number variation between individuals. We discarded short contigs (<1 Kb), contigs with low read counts across individuals, and male-biased contigs. Finally, we verified approximately 2-fold amplification of a subset of our X-linked regions in females as compared to males using qPCR.

Due in part to our conservative methods, we were limited in detecting the entire *Alouatta* X chromosome. Considering the combined length of the X-linked regions we detected is 26.2 Mb, it is likely they only partially represent the *A. palliata* X chromosome. This is not surprising given that our approach could not detect the pseudoautosomal region of the X chromosome (3 Mb in humans) since unlike the rest of the X chromosome, it is not hemizygous in males. We also expect that our dataset is an underrepresentation of the X chromosome given that much of the genome is repetitive, and we limited our analyses to non-repetitive sequences. Nonetheless, assuming the size of the *A. palliata* X chromosome is similar to the human and marmoset X chromosome and considering only the non-masked proportion of the human and marmoset X-chromosomal sequence (~59 Mb and ~57 Mb, respectively), the *A. palliata* sequences we identified to be X-linked here likely represent ~45% of the expected size of the non-masked *A. palliata* X chromosome. Thus, it is likely that our set of X-linked contigs will be built upon in future studies, and our results should be interpreted in this context.

### Conservation of the Mammalian X Chromosome in Alouatta

Because a large proportion of our X-linked contigs mapped to both the marmoset and human X chromosomes in similar positions, our results are consistent with the expectation of a high degree of sequence and gene order conservation in primates following high conservation of the eutherian mammal X chromosome (Quilter et al. 2002; Raudsepp et al. 2004; Murphy et al. 2007; Rodríguez Delgado et al. 2009). This result is also consistent with cytomolecular studies that identified a high degree of similarity between the human and *A. palliata* X chromosome using X chromosome paint probes (Steinberg et al. 2014). This is in contrast, however, to the high frequency of chromosomal rearrangements that have occurred in neotropical primates (Wienberg and Stanyon 1998; Müller 2006; de Oliviera et al. 2012) and suggests that despite the propensity for chromosomal rearrangements within *Alouatta*, including an autosome-Y translocation in *A. palliata* that forms a trivalent sex chromosome system (Solari and Rahn 2005; Steinberg et al. 2014), selection for conservation of the X chromosome in *A. palliata* may remain strong. Cytomolecular studies suggest the autosome-Y translocation is shared with *A. pigra* (Steinberg et al. 2014), but that *A. pigra* independently evolved an additional translocation (Steinberg et al. 2008), resulting in a quadrivalent sex chromosome system. The role these different sex chromosome systems play in reproductive isolation between these species has not been investigated, but we hypothesize that conservation of the X chromosome may provide the opportunity for meiotic recombination and ongoing gene flow between species. However, our finding of reduced introgression of some regions of the X chromosome suggests the sex chromosomes do play a role in reproductive isolation in this primate hybrid system.

Some of the female-biased contigs we identified in *A. palliata* mapped to marmoset autosomes (~26%). This pattern would be expected for autosomal regions that have been translocated from autosomes to the X chromosome in *Alouatta*. However, mapping positions for these contigs were shared and also common among male-biased and unbiased contigs. Thus, it is more likely that these regions are not unique to the X chromosome and represent false positives in our mapping experiment, or they represent marmoset X-chromosomal sequences that were erroneously mapped to autosomes. Investigation of what caused this putatively false-positive pattern and identification of any autosome-to-sex chromosome translocated regions are beyond the scope of this study and remain avenues for further research.

### X-Chromosomal Signature of Reproductive Isolation Shared With Humans

We found that, compared to autosomal SNPs, X-linked SNPs had reduced introgression (Figure 2). Regions with reduced introgression mapped to the long arm of the marmoset and human X chromosome in areas with a relatively low-density of genes (Supplementary Table S3). These results are consistent with our previous analyses on differential introgression in this system (Cortés-Ortiz et al. 2019), but because our previous analysis used both males and females (likely overinflating cline parameters for X-linked markers since males are hemizygous for the X chromosome), and only included 3 X-linked markers, this study provides more rigorous evidence for reduced introgression of the X chromosome in this system. We were also able to identify candidate regions of the *Alouatta* X chromosome that may underlie reproductive isolation due to the higher density of markers included in this study.

Reduced introgression of the X chromosome could result from a relatively high density of loci involved in reproductive isolation on the X chromosome and various other factors. For example, the presence of locally adapted alleles, which can accumulate more rapidly on the X chromosome than on autosomes (Charlesworth et al. 1987), would be expected to slow introgression of the X chromosome. Further, because the majority of the X chromosome only recombines in females, it has a reduced rate of recombination relative to autosomes (Schaffner 2004; Bohmanova et al. 2010), amplifying the effects of linkage around any adaptive alleles or barrier locus. Also, because males lack a second copy, the X chromosome has a relatively low effective population size (3/4 that of autosomes), making genetic drift more efficient and leading to reduced diversity and increased differentiation (Schaffner 2004). The effects of these factors are not expected to be mutually exclusive, and patterns of introgression do vary as a function of recombination rate and differentiation (Gompert et al. 2012; Janousek et al. 2015; Baiz et al. 2019). Because we do not have direct evidence linking specific loci to reproductive isolation mechanisms, we acknowledge that our finding of reduced introgression of sex chromosome markers in this system is indirect evidence for X-linked reproductive isolation (Presgraves 2018).

Reduced introgression of X-linked markers due to the presence of loci underlying reproductive isolation would be consistent with the large X-effect on postzygotic reproductive isolation (Coyne and Orr 1989). Anecdotal observations in this system indicate that hybrid males with intermediate admixture proportions (i.e., hybrid index ~0.5) may be sterile (L.C-O., personal observation). For example, we sampled a group in the hybrid zone multiple times containing a male with intermediate admixture proportions (Q = 0.46 based on SNP loci, Baiz et al. 2019), and even though he was the only reproductively mature male in the group for 7 years, no offspring were observed to be sired. Although this male is not an F1 individual (he carries the *A. pigra* haplotype for both mtDNA and *SRY*), he may carry combinations of incompatible alleles that hinder the production of sperm capable of fertilization.

It is interesting that one of our X-linked outliers with reduced introgression (region 3) falls within a known “desert” of both Neanderthal and Denisovan ancestry on the human X chromosome (Sankararaman et al. 2016), while the other (region 4) maps just distally (Figure 3). Sankararaman et al. (2016) hypothesized that hominin hybrid males suffered from reduced fertility because deserts of archaic ancestry on the human X chromosome are especially enriched near genes expressed in the testis. Because our region 3 outlier mapped to the central portion of one of these deserts, which spans a large section of the human X chromosome (34 Mb), we hypothesize that this window includes a region that underlies postzygotic reproductive isolation in both systems and thus may be important to the genetic architecture of speciation in primates. To further address this question, it will be highly informative to compare these observations to those from other primate systems. To our knowledge, the *Alouatta* system is the only natural non-human primate hybrid zone system that has been used specifically to identify genomic regions with candidate barrier loci. However, there are many known and genetically confirmed primate hybrid zone systems that could be used to similar ends (e.g., marmosets: Malukiewicz et al. 2015; chimpanzees: de Manuel et al. 2016; baboons: Wall et al. 2016; South American howlers: Mourthe et al. 2018). A recent study detected historical introgressive hybridization between bonobos and chimpanzees and found that gene exchange was restricted on the X chromosome (de Manuel et al. 2016). However, the authors did not report whether any X-linked regions were more or less resistant to introgression. Future studies on the genetics of hybridization and speciation in primates reporting such detail will allow for comparisons across studies and to address the hypothesis that a shared genetic architecture of reproductive isolation underlies speciation in primates.

### Directional Introgression of X Chromosome at Odds With Autosomes

In addition to a signature of reduced introgression, we found that X-linked SNPs tended to have excess ancestry from *A. palliata* more so than from *A. pigra.* Asymmetry in the direction of X chromosome introgression could be explained by a bias in the direction of backcrossing due to an unequal abundance of parental types in the hybrid zone or to directionality in prezygotic reproductive barriers (i.e., if hybrids carrying the *A. palliata* X chromosome are more likely to backcross with *A. pigra*, or *A. pigra*-like hybrids). Extensive sampling in this system has shown that both parental types, as well as multigenerational backcrossed hybrids into both parental types are relatively equally abundant (Kelaita and Cortés-Ortiz 2013; Cortés-Ortiz et al. 2019), suggesting that asymmetry in the direction of introgression is not caused by differences in abundance. If *A. pigra* males have traits more preferable to F1 females, then prezygotic barriers could explain the bias in X chromosome introgression. However, this is not consistent with the relatively equal abundance of backcrossed hybrid types, which indicates backcrossing occurs in both directions. Further, such bias due to the direction of backcrossing would be expected to carry over to autosomal loci. We found that autosomal loci do show asymmetry in the direction of introgression, but in the opposite direction—autosomal loci are enriched for *A. pigra* ancestry (Table 3, Supplementary Figure S6). Thus, these results may indicate that for the X chromosome, *A. palliata* alleles may be more favorable than *A. pigra* alleles in the hybrid zone when they do pass the species boundary, or that *A. pigra* alleles are less favorable in the habitat or genomic background of *A. palliata*. Outlier region 1 is particularly gene-rich (Table 4, Supplementary Table S3) and contains genes with varied functions, including functions related to the immune system (e.g., *FOXP3*, *WAS*, *CFP*), neuron function (e.g., *ELK1*, *SYN1*, *SYP*), and gene regulation (*FOXP3*, *SSX1*/*SSX4B*, *UXT*).

Because we used reduced-representation data in our genomic cline analysis, our ability to pinpoint regions that are driving the non-neutral patterns of introgression we observed is limited. Given that our genotype data represents a small portion of the genome, it is likely causal regions were not sequenced in our library and we are detecting effects of linkage to nearby genes that may be under selection in hybrid genomes. Future studies using whole-genome sequence data that represent the full scope of variation in these species will be needed to pinpoint candidate regions underlying non-neutral introgression.

## Conclusion

We identified extensive, contiguous X-chromosomal sequence in *A. palliata*, with regions exhibiting a high degree of conservation with the human and marmoset X chromosomes, consistent with conservation of the mammalian X chromosome. Our results revealed non-neutral introgression of the X chromosome in the *A. palliata* x *A. pigra* hybrid zone, consistent with a signature of reproductive isolation in some loci and with directional introgression in other loci.

Introgression of the X chromosome is reduced compared to autosomes, a genomic signature expected to occur as a result of postzygotic reproductive isolation. This is consistent with anecdotal evidence for hybrid male sterility in this system. Further, hybrid X chromosomes also exhibit an excess of *A. palliata* ancestry—a pattern that is opposite to autosomes, which exhibit an excess of *A. pigra* ancestry. Together, our results suggest that selection may be shaping introgression of the X chromosome and autosomes in different ways. Finally, one X-chromosomal region with significantly reduced levels of introgression overlaps a region of reduced archaic ancestry in the human genome, which suggests a shared genomic architecture of reproductive isolation in primates.

## Funding

This work was supported by the National Science Foundation (Division of Environmental Biology 0640519, Division of Behavioral and Cognitive Sciences 0962807 and 1517701 to L.C-O.); by a Grant‐In‐Aid of Research from the American Society of Mammalogists to M.D.B; a block grant from the Department of Ecology and Evolutionary Biology at the University of Michigan to M.D.B; by the University of Michigan Genetics Training Program (T32‐GM07544 to M.D.B.); and by National Institutes of Health (R01HD094736 to J. L. M.).

## Supplementary Material

esaa021_suppl_Supplementary_Material

## Data Availability

Whole-genome and reduced-representation sequence data used in this study are available from the NCBI Sequence Read Archive under accession numbers PRJNA553235 and PRJNA504885, respectively.

## References

[CIT0001] Baiz MD, Tucker PK, Cortés-Ortiz L. 2019. Multiple forms of selection shape reproductive isolation in a primate hybrid zone. Mol Ecol. 28:1056–1069. 30582763 10.1111/mec.14966 PMC6888905

[CIT0002] Barton NH, Hewitt GM. 1985. Analysis of hybrid zones. Annu Rev Ecol Syst. 16:113–148.

[CIT0003] Bohmanova J, Sargolzaei M, Schenkel FS. 2010. Characteristics of linkage disequilibrium in North American Holsteins. BMC Genomics. 11:421. 20609259 10.1186/1471-2164-11-421 PMC2996949

[CIT0004] Bracewell RR, Bentz BJ, Sullivan BT, Good JM. 2017. Rapid neo-sex chromosome evolution and incipient speciation in a major forest pest. Nat Commun. 8:1593. 29150608 10.1038/s41467-017-01761-4 PMC5693900

[CIT0005] Carling MD, Brumfield RT. 2008. Haldane’s rule in an avian system: using cline theory and divergence population genetics to test for differential introgression of mitochondrial, autosomal, and sex-linked loci across the Passerina bunting hybrid zone. Evolution. 62:2600–2615. 18691261 10.1111/j.1558-5646.2008.00477.x

[CIT0006] Carvalho AB, Clark AG. 2013. Efficient identification of Y chromosome sequences in the human and Drosophila genomes. Genome Res. 23:1894–1907. 23921660 10.1101/gr.156034.113 PMC3814889

[CIT0007] Charlesworth B, Coyne JA, Barton NH. 1987. The relative rates of evolution of sex chromosomes and autosomes. Am Nat. 130:113–146.

[CIT0008] Chen N, Bellott DW, Page DC, Clark AG. 2012. Identification of avian W-linked contigs by short-read sequencing. BMC Genomics. 13:183. 22583744 10.1186/1471-2164-13-183 PMC3428670

[CIT0009] Cortés-Ortiz L, Bermingham E, Rico C, Rodrıguez-Luna E, Sampaio I, Ruiz-Garcıa M. 2003. Molecular systematics and biogeography of the Neotropical monkey genus, Alouatta. Mol Phylogenetics Evol. 26:64–81. 10.1016/s1055-7903(02)00308-1 12470939

[CIT0010] Cortés-Ortiz L, Duda TF, Canales-Espinosa D, García-Orduña F, Rodríguez-Luna E, Bermingham E. 2007. Hybridization in large-bodied New World primates. Genetics 176:2421–2425. 17603105 10.1534/genetics.107.074278 PMC1950642

[CIT0011] Cortés-Ortiz, L, Nidiffer MD, Hermida-Lagunes J, García-Orduña F, Rangel-Negrín A, Kitchen DM, Bergman TJ, Dias PAD, Canales-Espinosa D. 2019. Reduced Introgression of Sex Chromosome Markers in the Mexican Howler Monkey (*Alouatta palliata × A. pigra*) Hybrid Zone. Int J Primatol. 40:114–131. 30880850 10.1007/s10764-018-0056-4 PMC6394575

[CIT0012] Coyne JA, Orr HA. 1989. Two rules of speciation. In: Otte D, Endler J, editors. Speciation and its consequences. Sunderland (MA): Sinauer Associates. p. 180–207.

[CIT0013] Danecek P, Auton A, Abecasis G, Albers CA, Banks E, DePristo MA, Handsaker RE, Lunter G, Marth GT, Sherry ST, 2011. The variant call format and VCFtools. Bioinformatics 27:2156–2158. 21653522 10.1093/bioinformatics/btr330 PMC3137218

[CIT0014] De Manuel M, Kuhlwilm M, Frandsen P, Sousa VC, Desai T, Prado-Martinez J, Hernandez-Rodriguez J, Dupanloup I, Lao O, Hallast P, 2016. Chimpanzee genomic diversity reveals ancient admixture with bonobos. Science 354:477–481. 27789843 10.1126/science.aag2602 PMC5546212

[CIT0015] de Oliveira EH, Neusser M, Müller S. 2012. Chromosome evolution in new world monkeys (Platyrrhini). Cytogenet Genome Res. 137:259–272. 22699158 10.1159/000339296

[CIT0016] Dobzhansky T. 1936. Studies on hybrid sterility. II. Localization of sterility factors in *Drosophila pseudoobscura* hybrids. Genetics. 21:113–135. 17246786 10.1093/genetics/21.2.113 PMC1208664

[CIT0017] Durinck S, Moreau Y, Kasprzyk A, Davis S, De Moor B, Brazma A, Huber W. 2005. BioMart and Bioconductor: a powerful link between biological databases and microarray data analysis. Bioinformatics 21:3439–3440. 16082012 10.1093/bioinformatics/bti525

[CIT0018] Durinck S, Spellman PT, Birney E, Huber W. 2009. Mapping identifiers for the integration of genomic datasets with the R/Bioconductor package biomaRt. Nat Protoc. 4:1184–1191. 19617889 10.1038/nprot.2009.97 PMC3159387

[CIT0019] Ellsworth JA, Hoelzer GA. 2006. Genetic evidence on the historical biogeography of Central American howler monkeys. In: Lehman SM, Fleagle JG, editors. Primate biogeography. Boston (MA): Springer. p. 81–103.

[CIT0020] Ford SM. 2006. The biogeographic history of Mesoamerican primates. In: Estrada A, Garber PA, Pavelka MSM, Luecke L, editors. New perspectives in the study of Mesoamerican primates. Boston (MA): Springer. p. 81–114.

[CIT0021] Gamble T, Coryell J, Ezaz T, Lynch J, Scantlebury DP, Zarkower D. 2015. Restriction site-associated DNA sequencing (RAD-seq) reveals an extraordinary number of transitions among gecko sex-determining systems. Mol Biol Evol. 32:1296–1309. 25657328 10.1093/molbev/msv023

[CIT0022] Gel B, Serra E. 2017. karyoploteR: an R/Bioconductor package to plot customizable genomes displaying arbitrary data. Bioinformatics. 33:3088–3090. 28575171 10.1093/bioinformatics/btx346 PMC5870550

[CIT0023] Gompert Z, Buerkle CA. 2011. Bayesian estimation of genomic clines. Mol Ecol. 20:2111–2127. 21453352 10.1111/j.1365-294X.2011.05074.x

[CIT0024] Gompert Z, Buerkle CA. 2012. *bgc*: Software for Bayesian estimation of genomic clines. Mol Ecol Resour. 12:1168–1176. 22978657 10.1111/1755-0998.12009.x

[CIT0025] Gompert Z, Lucas LK, Nice CC, Fordyce JA, Forister ML, Buerkle CA. 2012. Genomic regions with a history of divergent selection affect fitness of hybrids between two butterfly species. Evolution. 66:2167–2181. 22759293 10.1111/j.1558-5646.2012.01587.x

[CIT0026] Good JM, Dean MD, Nachman MW. 2008a. A complex genetic basis to X-linked hybrid male sterility between two species of house mice. Genetics. 179:2213–2228. 18689897 10.1534/genetics.107.085340 PMC2516092

[CIT0027] Good JM, Giger T, Dean MD, Nachman MW. 2010. Widespread over-expression of the X chromosome in sterile F_1_hybrid mice. PLoS Genet. 6:e1001148. 20941395 10.1371/journal.pgen.1001148 PMC2947990

[CIT0028] Good JM, Handel MA, Nachman MW. 2008b. Asymmetry and polymorphism of hybrid male sterility during the early stages of speciation in house mice. Evolution. 62:50–65. 18005156 10.1111/j.1558-5646.2007.00257.x PMC2907743

[CIT0029] Harris RS. 2007. Improved pairwise alignment of genomic DNA. Ph.D. Thesis, The Pennsylvania State University.

[CIT0030] Irwin DE. 2018. Sex chromosomes and speciation in birds and other ZW systems. Mol Ecol. 27:3831–3851. 29443419 10.1111/mec.14537

[CIT0031] Janoušek V, Munclinger P, Wang L, Teeter KC, Tucker PK. 2015. Functional organization of the genome may shape the species boundary in the house mouse. Mol Biol Evol. 32:1208–1220. 25631927 10.1093/molbev/msv011 PMC4408407

[CIT00310] Janoušek V, Wang L, Luzynski KEN, Dufková P, Vyskočilová MM, Nachman MW, Munclinger P, Macholán M, Piálek J, Tucker PK. 2012. Genome‐wide architecture of reproductive isolation in a naturally occurring hybrid zone between *Mus musculus musculus and M. m. domesticus.* Mol Ecol. 21:3032–3047. 22582810 10.1111/j.1365-294X.2012.05583.x PMC3872452

[CIT0032] Kelaita MA, Cortés-Ortiz L. 2013. Morphological variation of genetically confirmed *Alouatta pigra* × *A. palliata* hybrids from a natural hybrid zone in Tabasco, Mexico. Am J Phys Anthropol. 150:223–234. 23225250 10.1002/ajpa.22196

[CIT0033] Kitano J, Peichel CL. 2012. Turnover of sex chromosomes and speciation in fishes. Environ Biol Fishes. 94:549–558. 26069393 10.1007/s10641-011-9853-8 PMC4459657

[CIT0034] Li H, Handsaker B, Wysoker A, Fennell T, Ruan J, Homer N, Marth G, Abecasis G, Durbin R. 2009. The sequence alignment/map format and SAM tools. Bioinformatics 25:2078–2079. 19505943 10.1093/bioinformatics/btp352 PMC2723002

[CIT0035] Malukiewicz J, Boere V, Fuzessy LF, Grativol AD, e Silva IDO, Pereira LC, Ruiz-Miranda CR, Valença YM, Stone AC. 2015. Natural and anthropogenic hybridization in two species of eastern Brazilian marmosets (*Callithrix jacchus and C. penicillata*). PLoS One. 10:e0127268. 26061111 10.1371/journal.pone.0127268 PMC4464756

[CIT0036] Mendez FL, Poznik GD, Castellano S, Bustamante CD. 2016. The divergence of neandertal and modern human Y chromosomes. Am J Hum Genet. 98:728–734. 27058445 10.1016/j.ajhg.2016.02.023 PMC4833433

[CIT0037] Mongue AJ, Nguyen P, Voleníková A, Walters JR. 2017. Neo-sex chromosomes in the monarch butterfly, *Danaus plexippus*. G3-Genes Genom Genet. 7:3281–3294. 10.1534/g3.117.300187 PMC5633379 28839116

[CIT0038] Mourthe I, Trindade RA, Aguiar LM, Trigo TC, Bicca-Marques JC, Bonatto SL. 2018. Hybridization between neotropical primates with contrasting sexual dichromatism. Int J Primatol. 40:99–113.

[CIT0039] Muller HJ. 1942. Isolating mechanisms, evolution and temperature. Biol Symp. 6:71–125.

[CIT0040] Müller S. 2006. Primate chromosome evolution. In: Lupski JR, Stankiewicz P, editors. Genomic disorders: the genomic basis of disease. Totowa (NJ): Humana Press. p. 111–152.

[CIT0041] Murphy WJ, Davis B, David VA, Agarwala R, Schäffer AA, Wilkerson AJP, Neelam B, O’Brien SJ, Menotti-Raymond M. 2007. A 1.5-Mb-resolution radiation hybrid map of the cat genome and comparative analysis with the canine and human genomes. Genomics 89:189–196. 16997530 10.1016/j.ygeno.2006.08.007 PMC3760348

[CIT0042] Ohno S. 1967. Sex chromosomes and sex-linked genes. Berlin (Germany): Springer.

[CIT0043] Perelman P, Johnson WE, Roos C, Seuánez HN, Horvath JE, Moreira MA, Kessing B, Pontius J, Roelke M, Rumpler Y, 2011. A molecular phylogeny of living primates. PLoS Genet. 7:e1001342. 21436896 10.1371/journal.pgen.1001342 PMC3060065

[CIT0044] Peterson BK, Weber JN, Kay EH, Fisher HS, Hoekstra HE. 2012. Double digest RADseq: an inexpensive method for de novo SNP discovery and genotyping in model and non-model species. PLoS One. 7:e37135. 22675423 10.1371/journal.pone.0037135 PMC3365034

[CIT0045] Presgraves DC. 2008. Sex chromosomes and speciation in Drosophila. Trends Genet. 24:336–343. 18514967 10.1016/j.tig.2008.04.007 PMC2819171

[CIT0046] Presgraves DC. 2018. Evaluating genomic signatures of “the large X-effect” during complex speciation. Mol Ecol. 27:3822–3830. 29940087 10.1111/mec.14777 PMC6705125

[CIT0047] Quilter CR, Blott SC, Mileham AJ, Affara NA, Sargent CA, Griffin DK. 2002. A mapping and evolutionary study of porcine sex chromosome gene. Mamm Genome. 13:588–594. 12420137 10.1007/s00335-002-3026-1

[CIT0048] Raudsepp T, Lee EJ, Kata SR, Brinkmeyer C, Mickelson JR, Skow LC, Womack JE, Chowdhary BP. 2004. Exceptional conservation of horse–human gene order on X chromosome revealed by high-resolution radiation hybrid mapping. Proc Natl Acad Sci. 101:2386–2391. 14983019 10.1073/pnas.0308513100 PMC356960

[CIT0049] Robinson MD, McCarthy DJ, Smyth GK. 2010. edgeR: a Bioconductor package for differential expression analysis of digital gene expression data. Bioinformatics. 26:139–140. 19910308 10.1093/bioinformatics/btp616 PMC2796818

[CIT0050] Rodríguez Delgado CL, Waters PD, Gilbert C, Robinson TJ, Graves JAM. 2009. Physical mapping of the elephant X chromosome: conservation of gene order over 105 million years. Chromosome Res. 17:917–926. 19789986 10.1007/s10577-009-9079-1

[CIT0051] Sætre GP, Borge T, Lindroos K, Haavie J, Sheldon BC, Primmer C, Syvänen AC. 2003. Sex chromosome evolution and speciation in *Ficedula flycatchers*. Proc Royal Soc B. 270:53–59. 10.1098/rspb.2002.2204 PMC1691206 12590771

[CIT0052] Sankararaman S, Mallick S, Dannemann M, Prüfer K, Kelso J, Pääbo S, Patterson N, Reich D. 2014. The genomic landscape of Neanderthal ancestry in present-day humans. Nature 507:354–357. 24476815 10.1038/nature12961 PMC4072735

[CIT0053] Sankararaman S, Mallick S, Patterson N, Reich D. 2016. The combined landscape of Denisovan and Neanderthal ancestry in present-day humans. Curr Biol. 26:1241–1247. 27032491 10.1016/j.cub.2016.03.037 PMC4864120

[CIT0054] Schaffner SF. 2004. The X chromosome in population genetics. Nat Rev Genet. 5:43–51. 14708015 10.1038/nrg1247

[CIT0055] Smit AFA, Hubley R, Green P. *RepeatMasker Open-4.0.* 2013–2015 Available from: http://www.repeatmasker.org.

[CIT0056] Solari AJ, Rahn MI. 2005. Fine structure and meiotic behaviour of the male multiple sex chromosomes in the genus Alouatta. Cytogenet Genome Res. 108:262–267. 15545739 10.1159/000080825

[CIT0057] Steinberg ER, Nieves M, Mudry MD. 2014. Multiple sex chromosome systems in howler monkeys (Platyrrhini, Alouatta). Comp Cytogenet. 8:43–69. 24744833 10.3897/CompCytogen.v8i1.6716 PMC3978242

[CIT00570] Steinberg ER, Cortés-Ortiz L, Nieves M, Bolzán AD, García-Orduña F, Hermida-Lagunes J, Canales-Espinosa D, Mudry MD. 2008. The karyotype of *Alouatta pigra* (Primates: Platyrrhini): mitotic and meiotic analyses. Cytogenet Genome Res. 122:103–109. 19096205 10.1159/000163087

[CIT0058] Turner LM, Harr B. 2014. Genome-wide mapping in a house mouse hybrid zone reveals hybrid sterility loci and Dobzhansky-Muller interactions. Elife 3:e02504. 10.7554/eLife.02504 PMC4359376 25487987

[CIT0059] Vicoso B, Bachtrog D. 2015. Numerous transitions of sex chromosomes in Diptera. PLoS Biol. 13:e1002078. 25879221 10.1371/journal.pbio.1002078 PMC4400102

[CIT0060] Wall JD, Schlebusch SA, Alberts SC, Cox LA, Snyder‐Mackler N, Nevonen KA, Carbone L, Tung J. 2016. Genome-wide ancestry and divergence patterns from low‐coverage sequencing data reveal a complex history of admixture in wild baboons. Molecular Ecol. 25:3469–3483. 10.1111/mec.13684 PMC5306399 27145036

[CIT0061] Wienberg J, Stanyon R. 1998. Comparative chromosome painting of primate Genomes. ILAR J. 39:77–91. 11528067 10.1093/ilar.39.2-3.77

