# Supplementary material for: X-Linked Signature of Reproductive Isolation in Humans is Mirrored in a Howler Monkey Hybrid Zone
Source: J Hered. 2020 Jul 29;111(5):419–28. doi: 10.1093/jhered/esaa021 (PMC7525826; doi:10.1093/jhered/esaa021)
Supplement: esaa021_suppl_Supplementary_Material [file esaa021_suppl_supplementary_material.pdf]

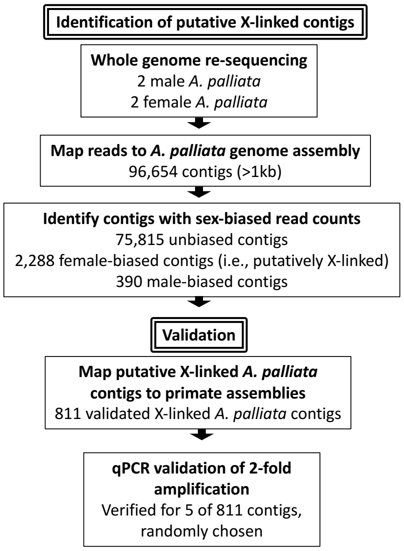

Figure S1. Overview of methods used in this study to identify and validate X-linkage for *A. palliata* assembly contigs.

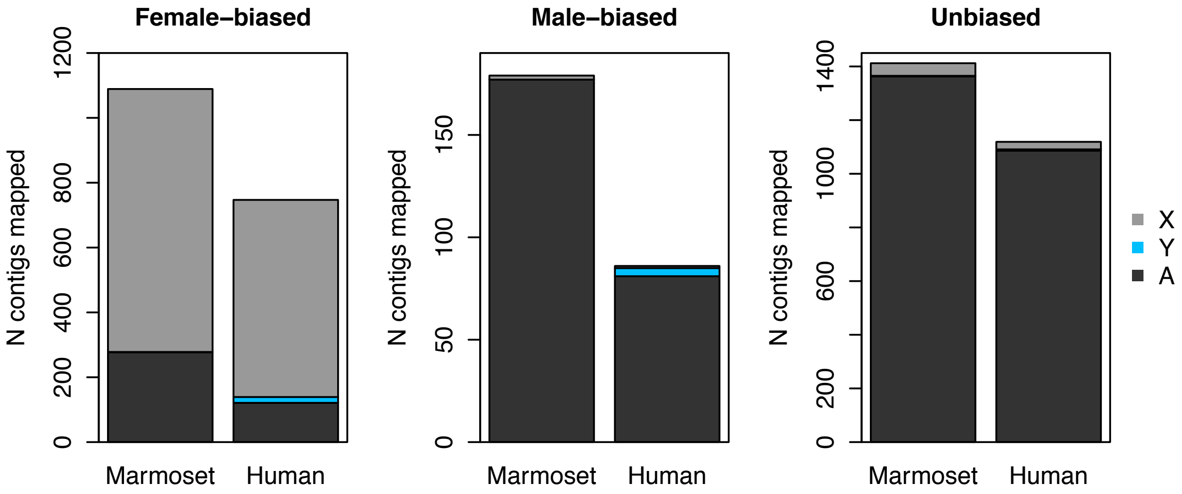

Figure S2. Number of contigs that mapped to the marmoset and human genome, for female-biased contigs, male-biased contigs, and unbiased contigs. Color denotes mapping position to either the X chromosome (light gray), the Y chromosome (blue), or to autosomes (dark gray).

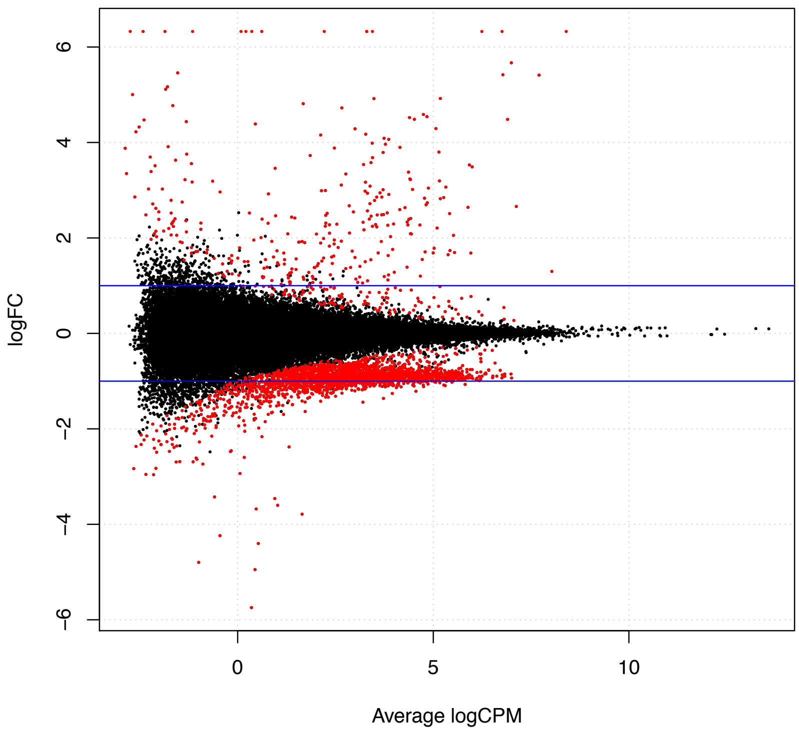

Figure S3. Summary of sex differences in read mapping count for *A. palliata* genome assembly contigs (N=96,654). Black dots represent contigs with no significant difference in read count between the sexes and are likely autosomal, while red dots represent contigs that show greater read counts for females (logFC<0) or males (logFC>0). Blue horizontal lines indicate a 2-fold difference in read count between the sexes. LogFC is log2-fold-change and Average logCPM is log2-counts-per-million, a measure of the number of reads mapped averaged across samples.

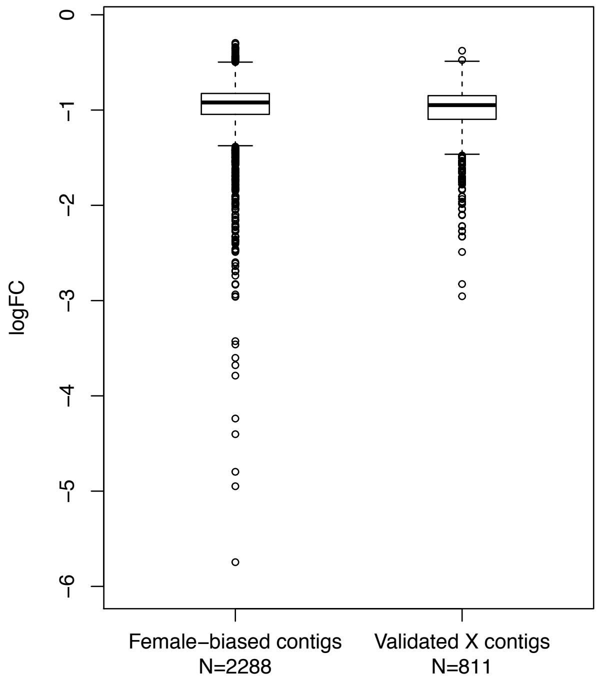

Figure S4. Male-to-female $\log_2$ fold-change in read mapping count for female-biased contigs across the genome and the subset of which mapped to the marmoset X chromosome (i.e., validated X contigs).

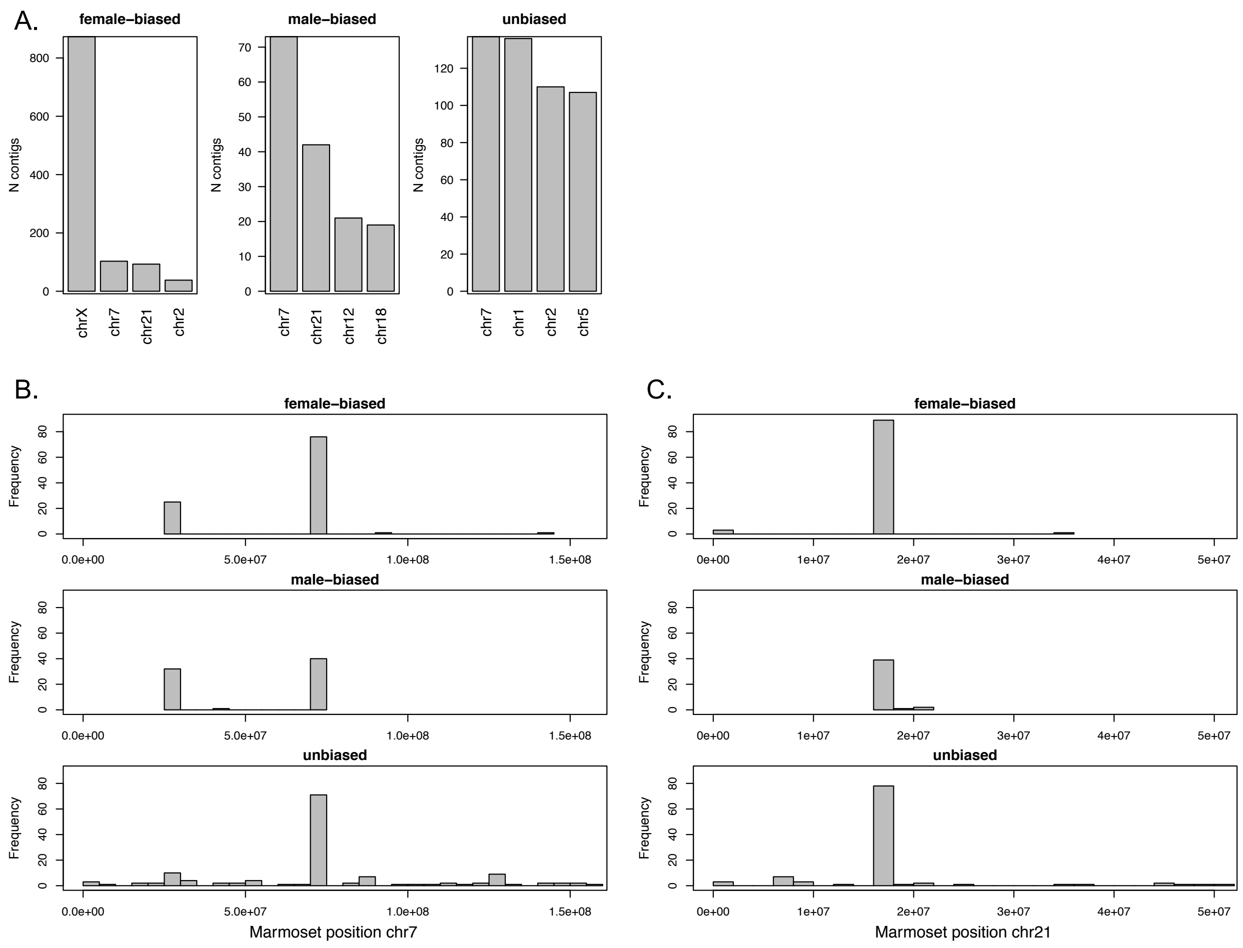

Figure S5. Mapping positions to the marmoset genome for sex-biased and unbiased contigs, for A) the top four chromosomes with most hits, B) chromosome 7, and C) chromosome 21. Panels B and C are histograms, where Frequency is the count of mapping positions.

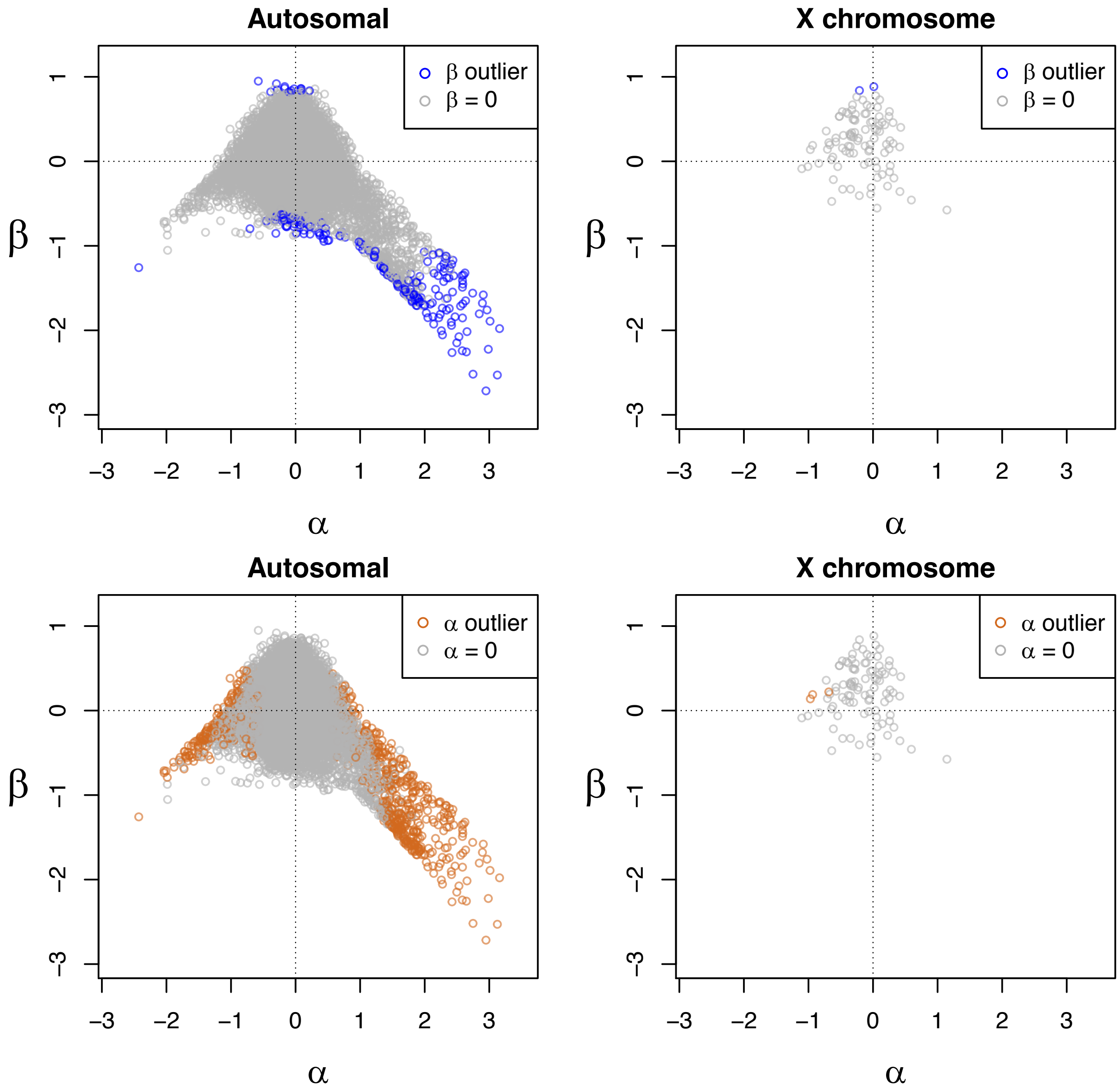

Figure S6. Cline parameter estimates for presumably autosomal SNPs (unbiased contigs, left panel) and X chromosomal SNPs (female-biased contigs, right panel). In the top panel, SNPs with a neutral amount (β) of introgression are gray and non-neutral amount (outliers) are blue. β>0=reduced introgression, β<0=increased introgression. In the bottom panel, SNPs with a neutral direction (α) of introgression are gray and non-neutral direction (outliers) are orange. α<0=excess *A. palliata* ancestry, α>0=excess *A. pigra* ancestry.

Table S1. Sample information for the *A. palliata* individuals used in the mapping experiment to identify X-linked contigs. Phenotypic sex = presumed sex based on visual assessment in the field, *SRY* = *SRY* (Y-linked gene) haplotype (NA = no amplification, Apm = *A. palliata* haplotype), *HAM80* = X-linked microsatellite genotype (allele sizes in bp). See Cortés-Ortiz et al. 2019 for details. N raw reads = number of paired reads obtained from the sequencer.

Sample ID	Phenotypic sex	*SRY* (Y)	*HAM80* (X)	N raw reads
S173	F	NA	138/138	89,770,052
S145	F	NA	132/136	123,076,486
S608	M	Apm	136	54,368,866
S618	M	Apm	132	90,755,417

Table S2. Primer information for *Alouatta* assembly contigs used in validation of X-linkage (A=autosomal contig, X=presumed X-linked contig, Marmoset position is the chromosomal coordinate for the start of the alignment block).

Contig	Contig length (bp)	Marmoset position	Primer sequences	Product length (bp)
84001^{A}	4,955	1: 182,469,879	ACGAATGCTTCAGGCTGAGT<br>AGGCAAGACCCACTGGTATG	163
26402^{X}	63,021	X: 104,175,832	CAGAGGCTAAATGGCTTTGC<br>TCTTGGCTGTTTGCATGAAG	102
35197^{X}	43,086	X: 83,726,463	CCCTCCCTGGAGAAAGAATC<br>CTTGGTTGCTTTGCAGATGA	113
92787^{X}	3,984	X: 52,026,941	TGCTTGTCATCCCAACACAT<br>GATTACAGACGCCCACCACT	144
118733^{X}	2,288	X: 6,638,526	ATGGGCTAGCAAGACTGCAT<br>TAGGAAGTGGGTTCCTGTGG	162
60023^{X}	14,492	X: 18,870,743	CACCTGTTGATGGACACTGG<br>GCCTTTGGCATTCAGATCAT	139

Table S3. Marmoset genes present in regions associated with *Alouatta* outlier X-linked SNPs that map to the human X chromosome. R=region as referred to in main text, calJac3=mapping position in the marmoset genome, Gene=HGNC symbol, Gene biotype=type of gene, Human homolog=orthologous human gene HGNC symbol, Human homolog %id=%id target human gene identical to marmoset gene, Human homolog type=number of marmoset genes relative to copies in the human genome, *bgc*=outlier type (α=direction of introgression: α>0=excess *A. pigra* ancestry, α<0=excess *A. palliata* ancestry. β=amount of introgression: β>0=reduced introgression, β<0=increased introgression).

R	calJac3	Gene	Gene biotype	Human homolog	Human homolog %id	Human homolog type	*bgc*
1	X:46524184:46524588		protein_coding				α<0
1	X:46559441:46570184	ARAF	protein_coding	ARAF	97.7	ortholog_one2one	α<0
1	X:46570165:46617942	SYN1	protein_coding	SYN1	98.9	ortholog_one2one	α<0
1	X:46583380:46587609		protein_coding				α<0
1	X:46601350:46601456		snRNA				α<0
1	X:46622530:46628236	CFP	protein_coding	CFP	87.6	ortholog_one2one	α<0
1	X:46634262:46645920	ELK1	protein_coding	ELK1	96.0	ortholog_one2one	α<0
1	X:46647105:46653594	UXT	protein_coding	UXT	97.6	ortholog_one2one	α<0
1	X:46783582:46856973	ZNF81	protein_coding	ZNF81	96.1	ortholog_one2one	α<0
1	X:46805656:46805760		rRNA				α<0
1	X:46912743:46943825	ZNF182	protein_coding	ZNF182	96.5	ortholog_one2one	α<0
1	X:46943345:47077150	SLC38A5	protein_coding	SLC38A5	90.2	ortholog_one2one	α<0
1	X:46944183:46949397		protein_coding	SPACA5	91.2	ortholog_one2many	α<0
1	X:46944183:46949397		protein_coding	SPACA5B	91.2	ortholog_one2many	α<0
1	X:47008402:47008505		snoRNA				α<0
1	X:47043931:47053167		protein_coding	SSX1	58.5	ortholog_one2many	α<0
1	X:47043931:47053167		protein_coding	SSX4	58.5	ortholog_one2many	α<0
1	X:47043931:47053167		protein_coding	SSX4B	58.5	ortholog_one2many	α<0
1	X:47043931:47053167		protein_coding	SSX3	58.0	ortholog_one2many	α<0
1	X:47043931:47053167		protein_coding	SSX7	57.4	ortholog_one2many	α<0
1	X:47043931:47053167		protein_coding	SSX5	56.4	ortholog_one2many	α<0
1	X:47043931:47053167		protein_coding	SSX2	48.4	ortholog_one2many	α<0
1	X:47043931:47053167		protein_coding	SSX2B	48.4	ortholog_one2many	α<0
1	X:47056142:47056261		rRNA	RNA5SP503	77.5	ortholog_one2many	α<0
1	X:47056142:47056261		rRNA	RNA5SP504	77.5	ortholog_one2many	α<0
1	X:47088308:47090170	FTSJ1	protein_coding	FTSJ1	94.8	ortholog_one2one	α<0
1	X:47116194:47129261	PORCN	protein_coding	PORCN	97.2	ortholog_one2one	α<0
1	X:47130218:47138922	EBP	protein_coding	EBP	96.5	ortholog_one2one	α<0
1	X:47152036:47171588	TBC1D25	protein_coding	TBC1D25	98.5	ortholog_one2one	α<0
1	X:47170685:47170785		snoRNA				α<0
1	X:47182903:47183225		pseudogene				α<0

1	X:47183584:47186730		protein_coding				α<0
1	X:47183584:47184464		pseudogene				α<0
1	X:47192501:47192596		misc_RNA	Y_RNA	84.4	ortholog_one2many	α<0
1	X:47210660:47223652	WDR13	protein_coding	WDR13	99.6	ortholog_one2one	α<0
1	X:47251356:47252178		protein_coding				α<0
1	X:47266935:47268993		pseudogene				α<0
1	X:47304180:47313473	WAS	protein_coding	WAS	94.6	ortholog_one2one	α<0
1	X:47305340:47305474		snoRNA				α<0
1	X:47310054:47310349		misc_RNA				α<0
1	X:47321721:47332971	SUV39H1	protein_coding	SUV39H1	94.7	ortholog_one2one	α<0
1	X:47396131:47412004	GLOD5	protein_coding	GLOD5	68.2	ortholog_one2one	α<0
1	X:47447336:47472570	HDAC6	protein_coding	HDAC6	91.4	ortholog_one2one	α<0
1	X:47478309:47479575	ERAS	protein_coding	ERAS	93.6	ortholog_one2one	α<0
1	X:47524542:47530403	TIMM17B	protein_coding	TIMM17B	98.8	ortholog_one2one	α<0
1	X:47530215:47535840	PQBP1	protein_coding	PQBP1	98.5	ortholog_one2one	α<0
1	X:47536135:47547272	SLC35A2	protein_coding	SLC35A2	97.4	ortholog_one2one	α<0
1	X:47548628:47554630	PIM2	protein_coding	PIM2	97.4	ortholog_one2one	α<0
1	X:47558863:47592157		protein_coding	OTUD5	94.0	ortholog_one2many	α<0
1	X:47594241:47594345		snRNA	RNU6-722P	86.7	ortholog_one2many	α<0
1	X:47597647:47602686	KCND1	protein_coding	KCND1	93.9	ortholog_one2one	α<0
1	X:47609181:47638593	GRIPAP1	protein_coding	GRIPAP1	78.2	ortholog_one2one	α<0
1	X:47631097:47631185		snoRNA	SNORA40	91.0	ortholog_one2one	α<0
1	X:47648691:47649616		pseudogene				α<0
1	X:47658718:47673447	TFE3	protein_coding	TFE3	87.4	ortholog_one2one	α<0
1	X:47691970:47703316	CCDC120	protein_coding	CCDC120	98.0	ortholog_one2one	α<0
1	X:47706609:47709461	PRAF2	protein_coding	PRAF2	91.5	ortholog_one2one	α<0
1	X:47710584:47716044	WDR45	protein_coding	WDR45	96.8	ortholog_one2one	α<0
1	X:47726915:47727499		pseudogene				α<0
1	X:47756195:47766273	GPKOW	protein_coding	GPKOW	91.4	ortholog_one2one	α<0
1	X:47781741:47781853		misc_RNA	Y_RNA	86.7	ortholog_one2one	α<0
1	X:47783521:47783814		misc_RNA	RN7SL262P	84.4	ortholog_one2one	α<0
1	X:47794754:47799714	MAGIX	protein_coding	MAGIX	90.1	ortholog_one2one	α<0
1	X:47804303:47807461	PLP2	protein_coding	PLP2	97.4	ortholog_one2one	α<0
1	X:47808048:47817687	PRICKLE3	protein_coding	PRICKLE3	91.2	ortholog_one2one	α<0
1	X:47817279:47817428		protein_coding				α<0
1	X:47830633:47840358	SYP	protein_coding	SYP	96.2	ortholog_one2one	α<0
1	X:47846758:47877754	CACNA1F	protein_coding	CACNA1F	95.3	ortholog_one2one	α<0
1	X:47866258:47866359		misc_RNA				α<0
1	X:47879139:47897726	CCDC22	protein_coding	CCDC22	95.1	ortholog_one2one	α<0
1	X:47898060:47912844		protein_coding	FOXP3	90.2	ortholog_one2one	α<0

1	X:47917171:47935050	PPP1R3F	protein_coding	PPP1R3F	92.3	ortholog_one2one	α<0
1	X:47949517:47951180		protein_coding				α<0
1	X:47949852:47949935		miRNA				α<0
1	X:47970163:48051622		protein_coding				α<0
1	X:47970163:48051622		protein_coding				α<0
1	X:47974726:47975839		pseudogene				α<0
1	X:48126558:48133698		protein_coding	PAGE1	42.1	ortholog_one2one	α<0
1	X:48298011:48302715	PAGE4	protein_coding	PAGE4	82.4	ortholog_one2one	α<0
1	X:48352872:48354800	USP27X	protein_coding	USP27X	72.6	ortholog_one2one	α<0
1	X:48398918:48566118	CLCN5	protein_coding	CLCN5	99.0	ortholog_one2one	α<0
1	X:48476370:48476460		miRNA				α<0
1	X:48476732:48476799		miRNA				α<0
1	X:48479887:48479964		miRNA				α<0
1	X:48481665:48481748		miRNA				α<0
1	X:48482210:48482287		miRNA				α<0
1	X:48483790:48483872		miRNA				α<0
2	X:67480740:67481444		protein_coding	MAGEE2	85.8	ortholog_one2one	α<0
2	X:67594298:67595150		protein_coding	ARL5A	79.1	ortholog_one2one	α<0
2	X:67696303:67701697		protein_coding	PBDC1	95.3	ortholog_one2many	α<0
2	X:68113106:68113192		miRNA				α<0
2	X:68288442:68288560		rRNA	RNA5SP509	91.6	ortholog_one2one	α<0
2	X:68480659:68481183		processed_pseudogene				α<0
3	X:114052741:114054497		protein_coding	DCAF12L2	94.4	ortholog_many2many	β>0
3	X:114052741:114054497		protein_coding	DCAF12L1	85.3	ortholog_many2many	β>0
3	X:114422500:114423885		protein_coding	DCAF12L2	83.3	ortholog_many2many	β>0
3	X:114422500:114423885		protein_coding	DCAF12L1	80.5	ortholog_many2many	β>0
3	X:114626321:114626427		snRNA				β>0
3	X:114657456:114659354	PRR32	protein_coding	PRR32	91.9	ortholog_one2one	β>0
3	X:114756176:114756222		miRNA				β>0
4	X:135646720:135647448		protein_coding				β>0
4	X:135691416:136093653		protein_coding	AFF2	97.4	ortholog_one2many	β>0
4	X:136008198:136008478		misc_RNA	RN7SKP267	85.1	ortholog_one2one	β>0
4	X:136141861:136179092		protein_coding	AFF2	89.4	ortholog_one2many	β>0

Supplementary methods

qPCR validation of X-linkage for A. palliata contigs

For qPCR validation, we designed a primer pair targeting each of the six contigs (5 putatively X-linked and one putatively autosomal contig, supplementary Table S2) using Primer 3 v. 0.4.0 (Koressaar and Remm 2007, Untergasser et al. 2012) using the 'human' setting to avoid designing primers in repetitive sequence, target size between 100-200bp, and otherwise default settings. We first verified amplification using standard PCR in at least three *A. palliata* individuals using the following cycling conditions: initial denaturation at 95ºC for 3 min, followed by 34 cycles of 95 ºC for 30 sec, annealing temperature of 55ºC for 30 sec, 72ºC for 30 sec, followed by 72ºC for 5 min. Amplifications were carried out using a reaction volume of 25 µl, containing 0.63 µl each of forward and reverse primer (10 µM), 0.125 µl GoTaq and 5 µl 5X green GoTaq buffer (Promega), 5 µl dNTPs (2µM each), 17.2 µl water, and 1 µl DNA extract. We included one negative control per reaction to ensure no contamination of our PCR reagents. We visualized PCR products on a 2% agarose gel to ensure amplification of a single band of the expected size.

For each primer pair, we then prepared 20 µl reactions for qPCR using 0.6 µl each of forward and reverse primer (10 µM), 10 µl Power SYBR Green PCR Master Mix (Applied Biosystems), 6.6 µl water, and 2.2 µl genomic DNA (~44 ng). For each primer set per run, we included three technical replicate amplifications each for one male and one female *A. palliata* individual. We also included one no-template DNA negative control per run using a single primer pair to ensure no contamination of PCR reagents. Amplifications were run on an ABI 7500 Fast Real-Time PCR machine with the following cycling conditions: 50ºC for 2 min, 95ºC for 10 min, followed by 40 cycles of 95ºC for 15 sec and 60ºC for 1 min. We calculated relative

fold-change in template DNA between the sexes using the $2^{-\Delta\Delta C}{}_{T}$ method (Livak and Schmittgen 2001), where the gene-of-interest was the presumed X-linked marker, the normalizing gene was the autosomal marker, and female was the ‘experimental’ condition while male was the ‘control’ condition.

References

Koressaar T, Remm M. 2007. Enhancements and modifications of primer design program Primer3. *Bioinformatics* 23:1289–91.

Livak KJ, Schmittgen TD. 2001. Analysis of relative gene expression data using real-time quantitative PCR and the $2^{-\Delta\Delta CT}$ method. *Methods* 25:402–408.

Untergasser A, et al. 2012. Primer3 - new capabilities and interfaces. *Nucleic Acids Res.* 40:e115
